# Metagenomic 16S rRNA gene sequencing survey of *Borrelia* species in Irish samples of *Ixodes ricinus* ticks

**DOI:** 10.1371/journal.pone.0209881

**Published:** 2019-04-15

**Authors:** John S. Lambert, Michael John Cook, John Eoin Healy, Ross Murtagh, Gordana Avramovic, Sin Hang Lee

**Affiliations:** 1 University College Dublin, Dublin, Ireland; 2 Mater Misericordiae University Hospital, Dublin, Ireland; 3 Vis a Vis Symposiums, Highcliffe, Dorset, United Kingdom; 4 University College Cork, Cork, Ireland; 5 Milford Molecular Diagnostics, Connecticut, United States of America; Cornell University, UNITED STATES

## Abstract

The spirochetal bacterium *Borrelia miyamotoi* is a human pathogen and has been identified in many countries throughout the world. This study reports for the first time the presence of *Borrelia miyamotoi* in Ireland, and confirms prior work with the detection of *B*. *garinii* and *B*. *valaisiana* infected tick*s*. Questing *Ixodes ricinus* nymph samples were taken at six localities within Ireland. DNA extraction followed by Sanger sequencing was used to identify the species and strains present in each tick. The overall rate of borrelial infection in the Irish tick population was 5%, with a range from 2% to 12% depending on the locations of tick collection. The most prevalent species detected was *B*. *garinii* (70%) followed by *B*. *valaisiana* (20%) and *B*. *miyamotoi* (10%). Knowledge of *Borrelia* species prevalence is important and will guide appropriate selection of antigens for serology test kit manufacture, help define the risk of infection, and allow medical authorities to formulate appropriate strategies and guidelines for diagnosis and treatment of *Borrelia* diseases.

## Introduction

There are more than 40 species in the genus *Borrelia*, currently divided into the Lyme borreliosis (LB) and Relapsing fever (RF) groups with human pathogens confirmed in both of these [1,2]. Whilst the major focus has been on Lyme disease borreliae the health burden of Relapsing fever (RF) species is now being recognized. *B*. *miyamotoi* classified in the RF group was first identified in Japan in 1995 [3] and subsequently found in many countries including the USA, Europe and England [4,5]. A prior study of *Borrelia* infections in ticks in Ireland identified three species, *B burgdorferi*, *B garinii*, *B valaisiana* (VS116) plus un-typeable *Borrelia* species [6].

In order to ensure detection of a wide range of *Borrelia* species that might be present in Ireland experiments were carried out to identify PCR primers for species that have been reported in Europe [2,7,8].

In the current study, we used a pair of genus-specific PCR primers to amplify a highly conserved segment with single-nucleotide polymorphisms of the borrelial 16S rRNA gene shared by all known pathogenic borrelial strains to survey the borrelial infections among the *I*. *ricinus* ticks collected in Ireland. Since this PCR amplicon is 357/358 bp long, the PCR products can be used as the template for direct Sanger sequencing for amplicon validation and for speciation [9]. This metagenomic 16S rRNA gene sequencing assay is most suitable for molecular diagnosis of borrelial infections in human-biting ticks and in clinical specimens in Europe because of the great diversity of causative agents in European Lyme borreliosis which needs a broad-spectrum tool to detect the target DNA from various borrelial strains and to prepare the template for Sanger sequencing to ensure diagnostic accuracy.

In the present study, we developed a protocol for using a single pair of genus-specific PCR primers to amplify a highly conserved segment with hypervariable regions of the borrelial 16S rRNA gene for detection of all species of *Borrelia* infecting the *I*. *ricinus* ticks collected in Ireland and to use the positive crude nested PCR products as the templates for direct Sanger sequencing to determine the species of the *Borrelia* detected.

## Materials and methods

### *Ixodes* tick collection and extraction

**Fig 1:**
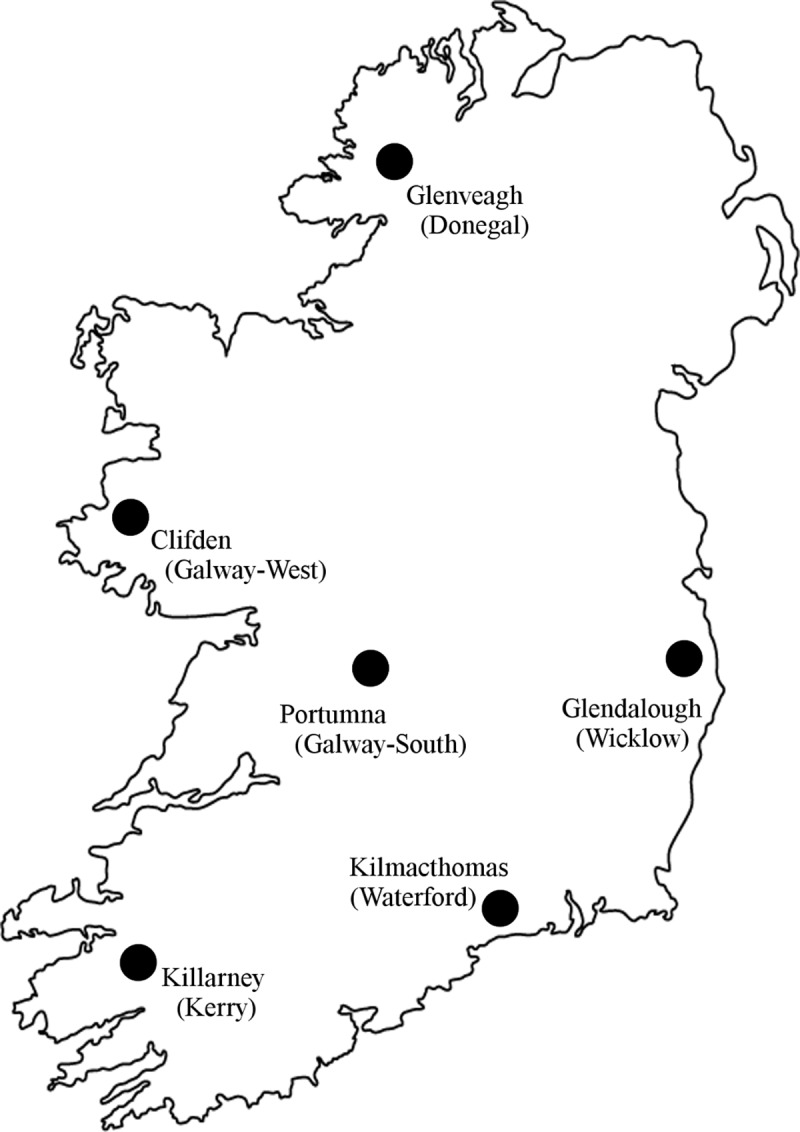
Sampling locations in Ireland for *Ixodes ricinus* ticks in May & June 2018.

Unfed, questing *I*. *ricinus* nymphs were collected by “flagging”, which involves brushing the vegetation with a white towel from which the ticks can then be removed. Nymphs were chosen for analysis since they occur in greater numbers than adult ticks, and pose the greatest risk to humans [10]. In late May and early June 2018, samples were taken at six localities within Ireland, designed to provide a representative view of tick borrelial infection across the country. The following areas were sampled (county in parenthesis): Killarney (Kerry), Kilmacthomas (Waterford), Clifden (Galway-West), Portumna (Galway-South), Glendalough (Wicklow), and Glenveagh (Donegal) and their locations are shown on the accompanying map of Ireland (Fig 1). No permission to access tick collection sites were required. All ticks were collected along the verges of public roads without requiring access to private land. There were no known endangered or protected species at any of the collection sites which all had public right of way access

Each individual sample was made up of at least 5 sub-samples taken at different points within a locality to minimize any sampling bias. Distance between sub-sampling points was never less than 100 m.

The collected ticks were disinfected in 70% ethanol, air-dried on filter paper and sent to Milford Molecular Diagnostics Laboratory in Milford, Connecticut, U.S.A. to be tested. The general procedure for extraction of the crude DNA from archived ticks and for Sanger sequencing detection of the borrelial 16S rRNA gene previously published [11] was followed. Initially, 300 ticks were analyzed, 50 from each of the six sampled locations listed above.

On the day of testing, each dried tick was placed in a 1.5 mL plastic tube and immersed in 300 μL of 0.7 mol/L NH_4_OH overnight at room temperature. On the following day, the test tubes were heated at 95°C to 98°C for 20 minutes with closed caps, followed by 10 minutes with open caps. After the test tubes were cooled to room temperature and the carcass of the tick discarded, 700 μL of 95% ethanol and 30 μL of 3 mol/L sodium acetate (Sigma) were added to each NH_4_OH digestate. The precipitated crude DNA was spun down in the pellet after centrifugation at ~ 16,000 x g for 5 minutes, washed in 1 mL of 70% ethanol, air dried, and re-dissolved in 100 μL of tris(hydroxymethyl)aminomethane hydrochloride–EDTA (TE) buffer, pH 7.4 (Sigma) by heating the DNA extract at 95°C to 98°C for 5 minutes. After a final centrifugation at ~ 16,000 x g for 5 minutes, the supernatant was used for molecular detection of borrelial 16S rRNA genes by nested PCR amplification.

### Molecular detection of borrelial 16S rRNA genes

A 3μL aliquot of the crude DNA extract was used to initiate a primary PCR, followed by a same-nested PCR using a pair of M1/M2 borrelial genus-specific primers in a total 25 μL low-temperature PCR mixture for a 30-cycle amplification at primary PCR, followed by another 30-cycle amplification at the same-nested PCR. In carrying out the same-nested PCR, a single pair of M1 (5'-ACGATGCACACTTGGTGTTAA-3') and M2 (5' TCCGACTTATCACCGGCAGTC-3') primers were used for both primary PCR and nested PCR so that a small amount of the primary PCR products was re-amplified with the same pair of PCR primers in a new PCR mixture [9]. An original target DNA segment in the PCR mixture might have been amplified for 60 cycles exponentially by the same pair of primers to increase the sensitivity of detection. The PCR amplicons of the 357-bp 16S rRNA gene segment of the *B*. *burgdorferi* sensu lato complex and the corresponding 358-bp 16S rRNA gene segment of the relapsing fever borreliae, both defined by the M1 and M2 PCR primer pair, were visualized by agarose gel electrophoresis of the nested PCR products. The same-nested PCR products were used as the template for Sanger reaction without purification.

A positive *Borrelia coriaceae* DNA and a negative (no extract) reagent control were routinely included in each run of our experiments, as detailed in a previously published reference [9]

### Sanger sequencing confirmation of *Borrelia* species

For DNA sequencing, the positive nested PCR products were transferred by a micro-glass rod into a Sanger reaction tube containing 1 μL of 10 μmolar sequencing primer, 1 μL of the BigDye Terminator (v 1.1/Sequencing Standard Kit), 3.5 μL 5× buffer, and 14.5 μL water in a total volume of 20 μL for 20 enzymatic primer extension/termination reaction cycles according to the protocol supplied by the manufacturer (Applied Biosystems, Foster City, CA, USA). After a dye-terminator cleanup with a Centri-Sep column (Princeton Separations, Adelphia, NJ, USA), the reaction mixture was loaded in an automated ABI 3130 four-capillary Genetic Analyzer for sequence analysis.

Sanger sequencing of positive 357/358 bp M1/M2 same-nested PCR products is capable of accurate identification of many species including, *B*. *valaisiana*, *B*. *afzelii*, *B*. *mayonii*, *B*. *spielmanii*, *B*. *lusitaniae*, *B*. *recurrentis*, *B*. *miyamotoi*, *B*. *hermsii*, *B*. *lonestari*, *B*. *coriaceae* and several other members in the relapsing fever group based on known species-specific single-nucleotide polymorphisms in the gene segment [9].

The “genus-specific” M1/M2 PCR primer pair can amplify a “core genome” of all pathogenic borreliae for the purpose of detection. However, to design a pair of reliable PCR primers to amplify a segment of borrelial 16S rRNA gene with single-nucleotide polymorphism among various borrelial strains turned out to be challenging. It took several weeks of experimental work before we found 3 PCR primers to generate a 282-bp heminested PCR amplicon useful as the template for Sanger sequencing to distinguish *B*. *garinii* from *B*. *burgdorferi* and to discriminate among the various *B*. *garinii* strains. The sequences of these 3 heminested PCR primers are listed as follows.

Primary PCR Forward Primer Bg1: 5’- GACGTTAATTTATGAATAAGC -3’

Primary PCR Reverse Primer Bg 6: 5’- TTAACACCAAGTGTGCATCGT– 3’

Heminested PCR Forward Primer Bg5: 5’- CGGGATTATTGGGCGTAAAGGGTGAG-3’

Heminested PCR Reverse Primer Bg 6: 5’- TTAACACCAAGTGTGCATCGT– 3’

Reference sequences were retrieved from the GenBank, the Bg5/Bg6 heminested PCR primer pair defines a 282 bp segment of the borrelial 16S rRNA gene with single-nucleotide polymorphisms. These were used to discriminate *Borrelia burgdorferi* strain B31 (ID# CP019767), *Borrelia garinii* BgVir (ID# CP003151), *Borrelia garinii* strain Bernie (ID# D89900), *Borrelia garinii* strain T25 (ID# AB035388) and *Borrelia garinii* strain L20 (ID# X85198).

However, after the first 300 ticks were tested, it was realized that the extracellular DNA of the *Borrelia* 16S rRNA gene in the crude DNA extract from the ticks was not stable on storage even at -20° C. By the time when the Bg5 and Bg6 heminested PCR primers were readied to be put into routine practice, the borrelial 16S rRNA gene DNA in 9 of the 12 samples initially found to be positive for *B*. *burgdorferi* sensu lato already degraded and were no longer amplifiable with any PCR primers. Therefore, an additional series of 50 ticks from each of the Portumna and Kilmacthomas samples were analyzed for the specific purpose of species confirmation and strain determination of the *B*. *garinii* isolates.

## Results

### Multiple *Borrelia* species found in *I*. *ricinus* ticks in Ireland

**Fig 2:**
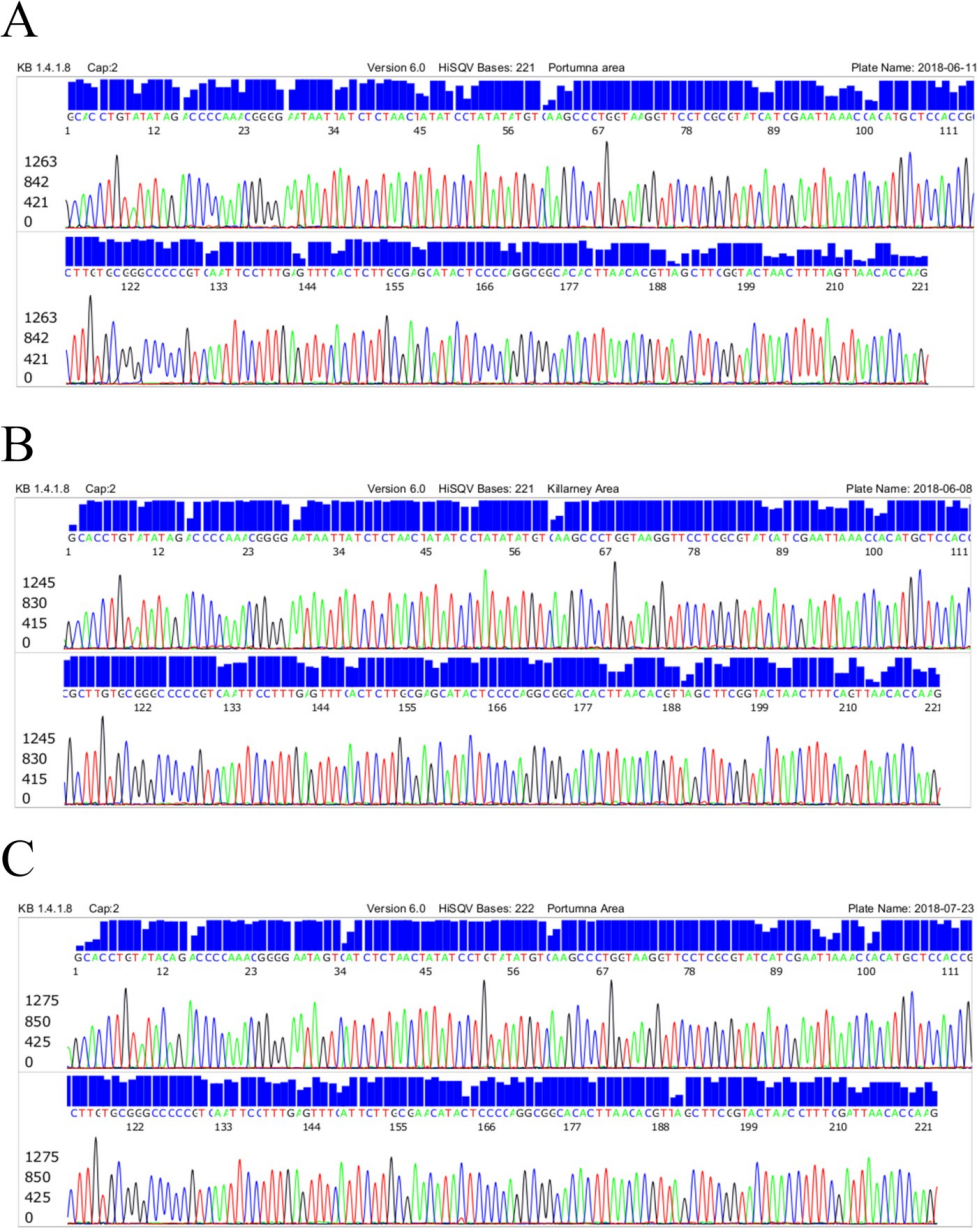
Samples of computer-generated electropherograms of 16S rRNA gene sequences representing the 3 borrelial groups detected in the ticks collected in Ireland. (A) 221 bases Portumna area–*B*. *burgdorferi* sensu lato (GenBank ID# CP019767). (B) 221 bases Killarney Area–*B*. *valaisiana* (GenBank ID# AB091815). (C) 222 bases Portumna Area–*B*. *miyamotoi* (GenBank ID# KM007554).

The same-nested PCR amplification of a 357/358 bp segment of borrelial 16S rRNA gene by the M1/M2 genus-specific PCR primers followed by Sanger sequencing of the nested PCR products provided metagenomic evidence of *B*. *burgdorferi* sensu lato (B.b.s.l.), *B*. *valaisiana* and *B*. *miyamotoi* infection in the ticks collected in Ireland. Samples of the 16S rRNA gene sequencing with the M2 primer in support of these molecular diagnoses are illustrated by the 3 selected electropherograms presented in Fig 2.

### Strain diversity of *B*. *garinii*

Sanger sequencing of the PCR amplicon with the reverse M1 primer proved that none of the *B*. *burgdorferi* sensu lato isolates were *B*. *afzelii*. A 177-base sequence of the 282-bp amplicon defined by the Bg5 and Bg6 heminested PCR primers distinguished the heterogeneous strains of *B*. *garinii* from *B*. *burgdorferi* and *B*. *bavariensis* due to the presence of single-nucleotide polymorphisms among strain BgVir {1}, strain 25 {2} and strain Bernie {3} of the *B*. *garinii* species and distinguished these strains from *B*. *burgdorferi* sensu stricto {4}. Alignment of these four 177-base reference sequences retrieved from the GenBank showing single-nucleotide polymorphisms is presented below with the ending 26-base Bg5 primer site underlined.

{1}CCTTCGCCTCCGGTATTCTTTCTGATATCAACAGATTCCACCCTTACACCAGAAATTCTAACTTCCTCTATCAGACTCTAGAC

{2}··················································································· {3}··················································································T

{4}···················································································

{1}ATATAGTTTCCAACATAGTTCCACAGTTGAGCTGTGGTATTTTATGCATAGACTTATATATCCGCCTACTCACCCTTTACGCC

{2}································C····C·············································

{3}···················································································

{4}··················G································································

{1}CAATAATCCCG *B*. *garinii* Strain BgVir Sequence ID: CP003151 (Range:447801–447977)

{2}··········· *B*. *garinii* Strain T25 Sequence ID: AB035388 (Range: 684–508)

{3}··········· *B*. *garinii* Strain Bernie Sequence ID: D89900 (Range:692–516)

(4}··········· *B*. *burgdorferi* strain B31 Sequence ID: CP019767 (Range: 444582–444758)

**Fig 3:**
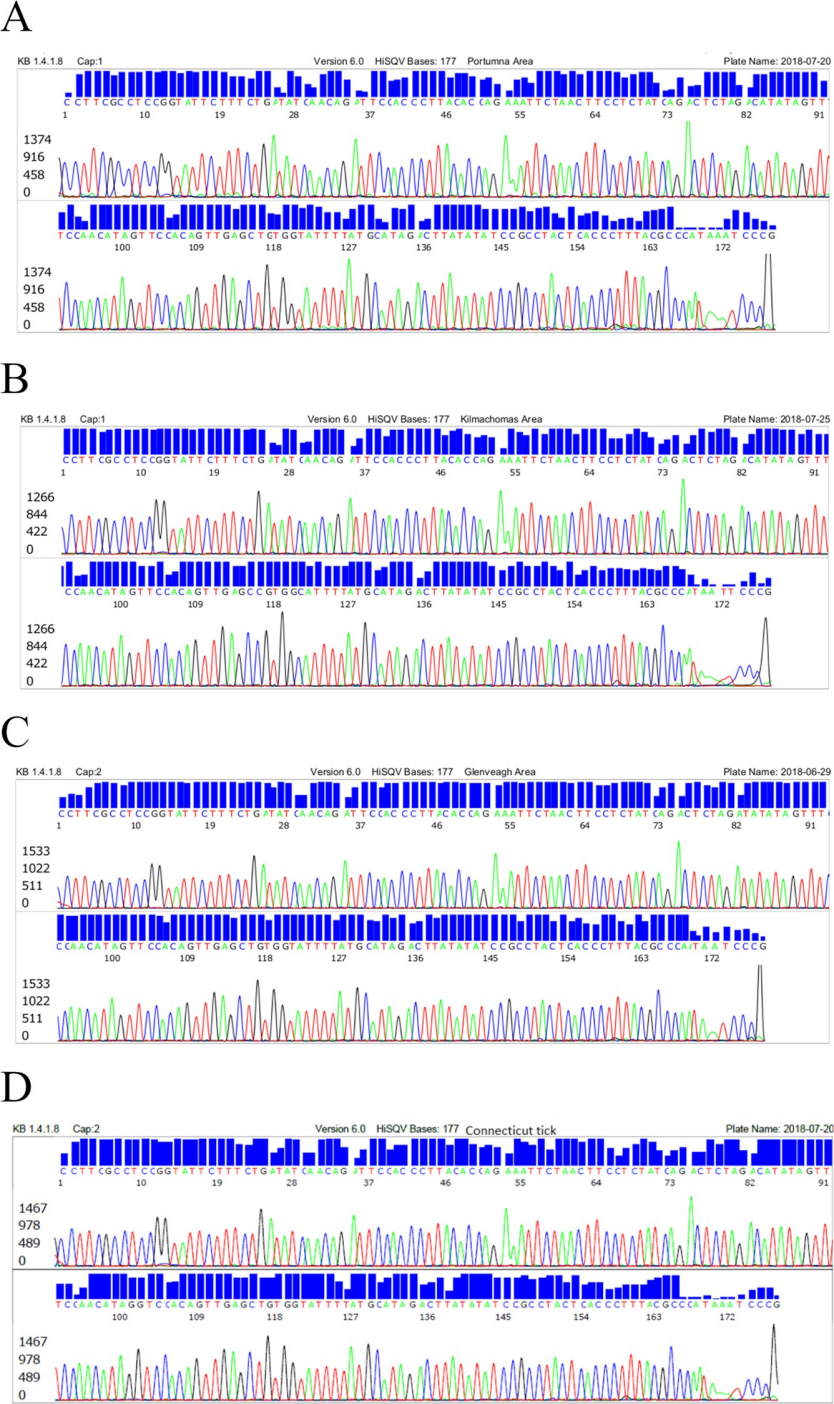
DNA sequencing electropherograms for distinguishing *B*. *garinii strains*. (A) Strain BgVir. (B) Strain T25. (C) Strain Bernie. (D) *B*. *burgdorferi* strain B31.

The corresponding electropherograms of these four 177-base sequences (1–4), using the Bg6 sequencing primer, are illustrated in Fig 3, arranged from top to bottom in numerical order of sequences 1–4 as Strain BgVir, Strain T25, Strain Bernie and *B*. *burgdorferi* strain B31.

A reverse sequencing with Bg5 primer of the 282-bp PCR amplicon confirmed that the isolates identified as strains BgVir were not strain SL20 (Sequence ID: X85198) because there is no single-nucleotide polymorphism located near the Bg6 primer site of the amplicon which is unique for strain SL20.

### Tick infection rates varied with locations

We conducted two surveys. Survey 1 consisted of 300 ticks collected in six locations showing 15 of the 300 ticks (5%) infected with *B*. *burgdorferi* sensu lato of which 3 were confirmed to be *B*. *garinii* (2 matching strain Bernie and 1 matching strain BgVir), 2 were *B*. *valaisiana* and 10 were not speciated. Survey 2 consisted of 100 ticks collected in two of the six locations previously surveyed, showing 5 of the 100 ticks (5%) infected by borrelial species, including 4 isolates of *B*. *garinii* (3 matching strain BgVir and 1 matching strain T25) and 1 isolate of *B*. *miyamotoi*. The *Borrelia* infection rates varied from 2% to 12% depending on the locations of the tick collection. It is noteworthy that all *B*. *burgdorferi* sensu lato isolates in Survey 2 were confirmed to be *B*. *garinii* as the Bg5/BG6 PCR followed by Sanger sequencing for speciation was carried out without delay after initial detection by M1/M2 PCR. The final species distributions in different locations were summarized in Table 1.

**Table 1:** Incidence of Borrelia species in Irish *Ixodes ricinus* tick samples(nymphs).

Location	Sample size	No. Positive	% Positive	CI 95%	Borrelia species
Killarney (Kerry)	50	1	2%	(0% - 6%)	*B*. *valaisiana*
Kilmacthomas (Waterford)	50	3	6%	(0% - 13%)	*3x B*.*b s*.*l*
Kilmacthomas (Survey 2)	50	2	4%	(0% - 9%)	*2x B*. *garinii*
Portumna (Galway-South)	50	6	12%	(3% - 21%)	*6x B*.*b s*.*l*
Portumna (Survey 2)	50	3	6%	(0% - 13%)	*1x B*. *miyamotoi 2x B*. *garinii*
Glendalough (Wicklow)	50	1	2%	(0% - 6%)	*B*. *valaisiana*
Glenveagh (Donegal)	50	3	6%	(0% - 13%)	*3x B*. *garinii*
Clifden (Galway-West)	50	1	2%	(0% - 6%)	*B*.*b s*.*l*
Total	400	20	5%	(3% - 7%)	
		Of 10 speciated infected ticks:			
		*B*. *garinii*	70%	(7/10)	
		*B*. *miyamotoi*	10%	(1/10)	
		*B*. *valaisiana*	20%	(2/10)	
Percent other *B*.*b*.*s*.*l*. not speciated			50%	(10/20)	
					

*B*.*b s*.*l = Borrelia burgdorferi sensu lato*. CI 95% = 95% confidence interval

### Selection of PCR primers for 16S rRNA gene PCR and sequencing

**Fig 4:**
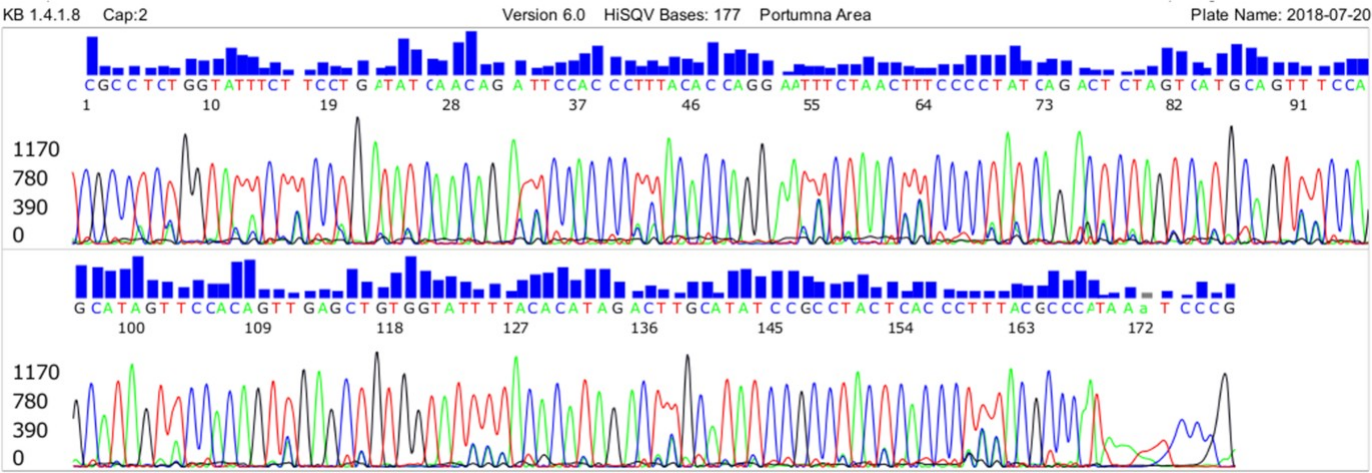
DNA sequencing of Bg5/Bg6 primer nested PCR products of *B*. *miyamotoi* 16S rRNA gene.

We used the “genus-specific” M1/M2 primer pair to generate a PCR amplicon for Sanger reaction to detect all species of the B.b.s.l. complex and the relapsing fever borreliae, in particular *B*. *miyamotoi*. And a heminested PCR system to amplify an adjacent 282 bp segment defined by the Bg5 and Bg6 primers (Fig 3). When the Bg5/Bg6 nested PCR products were used as the DNA sequencing template for confirmation of *B*. *miyamotoi* in tick samples, the electropherograms showed numerous ambiguous base calling peaks as a result of co-amplification of unwanted DNAs in the sample extract (Fig 4). When the sequencing electropherogram generated by the M1/M2 primer PCR products from the same tick extract showed no ambiguous base calling labels (Fig 2C).

### Degradation of 16S rRNA gene DNA in samples

**Fig 5:**
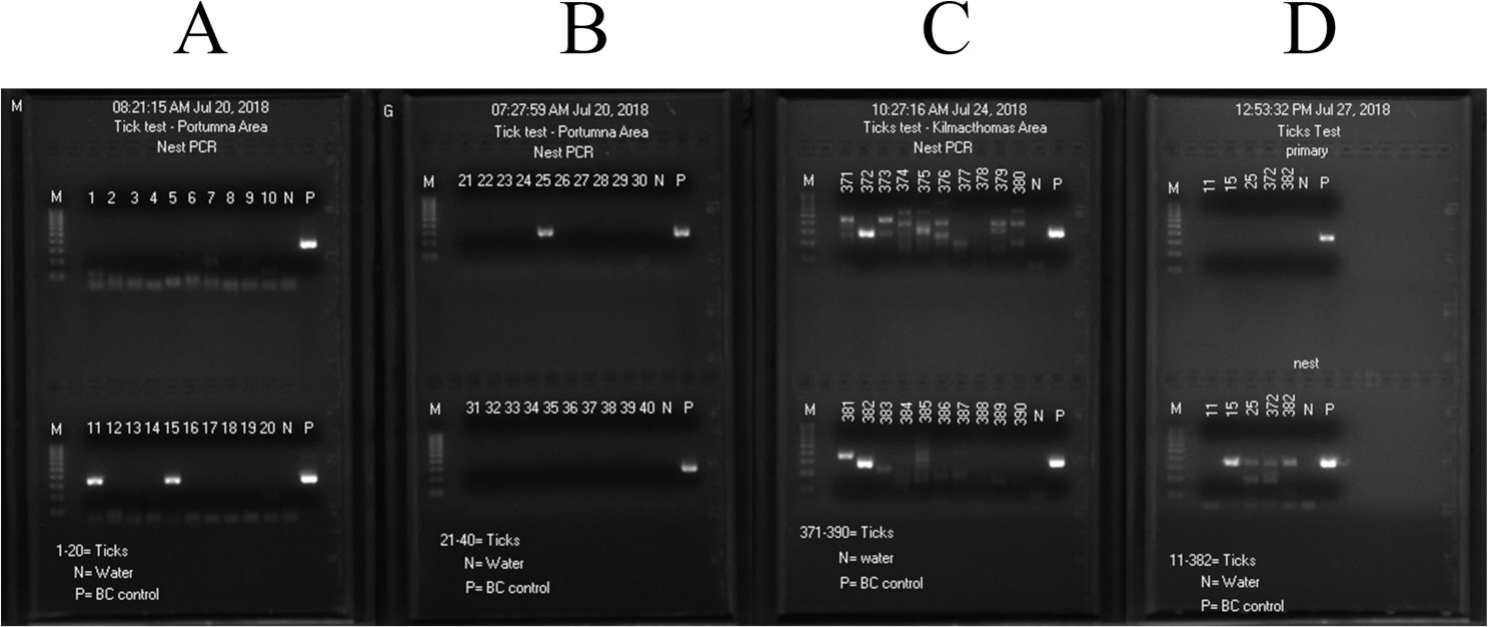
Images of agarose gel electrophoresis. Images on July 20 and July 24, 2018 (A-C) show positive 357/358 bp borrelial 16S rRNA gene nested PCR amplicons in five positive tick samples on the day of DNA extraction (Lanes 11, 15, 25, 372 and 382). After storage of the DNA extracts at -20°C, the same DNA preparations were used to repeat primary and nested PCR on July 27, 2018 (D), showing no nested PCR products for sample 11; the amount of nested PCR products on samples 15, 25, 372 and 382 was significantly reduced. N = negative control; P = positive *Borrelia coriaceae* control. M = molecular ruler. Note numerous non-specific PCR amplifications on panel for samples 371–390.

We also observed that free borrelial 16S rRNA gene DNA in crude extracts from the ticks was unstable even in TE buffer stored at -20°C for an extended period of time. For example, when the NH_4_OH extracts of the 50 ticks from the Portumna area were processed for M1/M2 same-nested PCR screening on July 20, 2018, 3 samples were positive for borrelial 16S rRNA gene DNA, with a robust 357/358 bp band on lanes 11, 15 and 25 (Fig 5) which eventually proved to be *B*. *garinii* strain BgVir, *B*. *miyamotoi* and *B*. *garinii* BgVir, respectively. When the NH_4_OH extracts of the 50 ticks from the Kilmacthomas area were processed for M1/M2 same-nested PCR screening on July 24, 2018, 2 samples were positive for borrelial 16S rRNA gene DNA, with a robust 357/358 bp band on lanes 372 and 382 (Fig 5) which eventually proved to be *B*. *garinii* with sequence matching strain BgVir and *B*. *garinii* strain T25, respectively. However, when the same-nested PCR was repeated on these 5 NH_4_OH extracts after 7 days and 3 days storage in a -20°C freezer, respectively, the 16S rRNA gene DNA in sample 11 was no longer detectable and the intensities of the nested PCR bands using the same extracts of samples 15, 25, 372 and 382 as primary PCR templates for amplification under identical experimental conditions decreased markedly over a period of 3–7 days, as demonstrated on the agarose gel dated July 27, 2018 (Fig 5). The image of the gel electrophoresis dated July 27, 2018 also showed that nested PCR is generally required for detection of borrelial infections by 16S rRNA gene analysis. Primary PCR products are usually invisible after gel electrophoresis.

## Discussion

This is the first time that *B*. *miyamotoi* has been detected in a tick collected in Ireland.

Our study shows that *I*. *ricinus* ticks in Ireland are infected by a diversity of pathogenic borreliae. The number of species and strain diversity may prove to be greater if surveys with larger samples are carried out. For example, in our two surveys *B*. *miyamotoi* and *B*. *garinii* were demonstrated only during Survey 2, but not in Survey 1 (Table 1). Our results demonstrate that *Borrelia*-infected tick populations exist in the south-east of Ireland, an area hitherto not considered to be significantly tick-infested and hence not considered an area of risk to humans for contraction of Lyme borreliosis.

In the prior work carried out by Kirstein et al tick infection rates were reported between 3.5% and 26.7% depending on the locations of tick collection using reverse line blot or PCR [6]. In the current study, we used metagenomic 16S rRNA gene sequencing to survey the *I*. *ricinus* ticks, and found the overall rate of borrelial infection to be 5%, with a range from 2% to 12% depending on the locations of tick collection (Table 1). In Glenveagh (Donegal) there were two different strains of *B*. *garinii* with sequences that matched BgVir, and Bernie). This compares to infected nymphs collected in Glenveagh National Park 20 years earlier which were reported to be positive for *B*. *valasiana* (labeled as strain VS116). We identified *B*. *valaisiana* in Gelendalough and Killarney, and *B*. *miyamoti* plus *B*. *garinii* in Portumna.

For the purpose of patient care, it is probably not necessary to determine the species or strain of the *Borrelia* infection in order to initiate timely antibiotic treatment. But, for serological diagnosis a knowledge of the borrelial species and strains carried by the human-biting ticks collected in the endemic areas is crucial since polymorphism of ospC [12–14], and variation of the VlsE antigens among species and strains of *B*. *burgdorferi* sensu lato [15] have been well documented in the literature. There is limited information regarding the sensitivity of commercial tests for different species of *Borrelia*. The manufacturers of Western Blot antibody tests specify the target species. In Europe these are typically *B*. *afzelii*, *B*. *burgdorferi* and *B*. *garinii*. A study of the most frequently used C6 synthetic peptide ELISA test for initial screening does appear to have variable sensitivity for some species [16] but with no data available for many of the European species including *B*. *valaisiana* identified as prevalent in Ireland in this study.

In summary, our study confirms that the genus-specific M1/M2 PCR primers can amplify a highly conserved segment of the borrelial 16S rRNA gene for Sanger sequencing-based molecular diagnosis of tick-borne borreliae. Three species of *Borrelia* were identified with *B*. *garinii* the most common (70% of those speciated), followed by *B*. *valaisiana* (20%), and for the first time in Ireland *B*. *miyamotoi* (10%). This study and future expanded surveillance of the Borrelia species and prevalence will contribute to optimized testing for patients and to help quantify the risks and potential burden of human *Borrelia* infections in Ireland.

## Supporting information

S1 Table Minimum data set showing Tick infection rates and Confidence Interval calculations. (XLSX)
